# Myotonic dystrophies: an update on clinical features, molecular mechanisms, management, and gene therapy

**DOI:** 10.1007/s10072-024-07826-9

**Published:** 2024-12-07

**Authors:** Martina Rimoldi, Sabrina Lucchiari, Serena Pagliarani, Giovanni Meola, Giacomo Pietro Comi, Elena Abati

**Affiliations:** 1 Neurology Unit, IRCCS Fondazione Ca’ Granda Ospedale Maggiore Policlinico, Milan, Italy; 2 Medical Genetic Unit, IRCCS Fondazione Ca’ Granda Ospedale Maggiore Policlinico, Milan, Italy; 3 Department of Pathophysiology and Transplantation (DEPT), Neuroscience Section, Dino Ferrari Centre, University of Milan, Milan, Italy; 4 Department of Biomedical Sciences for Health, Department of Neurorehabilitation Sciences, University of Milan, Casa di Cura Igea, Fondazione Malattie Miotoniche -FMM, Milan, Italy

**Keywords:** DM1, DM2, Myotonic dystrophies, Molecular genetics, Management, Update

## Abstract

Myotonic dystrophies (DM) encompass a group of complex genetic disorders characterized by progressive muscle weakness with myotonia and multisystemic involvement. The aim of our paper is to synthesize key findings and advancements in the understanding of DM, and to underline the multidisciplinary approach to DM, emphasizing the importance of genetic counseling, comprehensive clinical care, and symptom management. We discuss the genetic basis of DM, emphasizing the role of repeat expansions in disease pathogenesis, as well as cellular and animal models utilized for studying DM mechanisms and testing potential therapies. Diagnostic challenges, such as determining the size of disease expansions and assessing mosaicism, are elucidated alongside emerging genetic testing methods. Therapeutic strategies, mainly for DM1, are also explored, encompassing small molecules, nucleic acid-based therapies (NATs), and genome/transcriptome engineering. The challenges of such a therapeutic delivery and immunogenic response and the importance of innovative strategies, including viral vectors and AAV serotypes, are highlighted within the text. While no curative treatments have been approved, supportive and palliative care remains essential, with a focus on addressing multisystemic complications and maintaining functional independence. Continued exploration of these therapeutic advancements offers hope for comprehensive disease management and potentially curative therapies for DM1 and related disorders.

## Introduction

Myotonic dystrophies are the most common muscular dystrophy in adults, autosomal inherited, mainly characterized by muscle weakness and myotonia, with multisystemic involvement. So far 2 distinct entities have been described: Myotonic Dystrophy type 1 (DM1) (OMIM #160900) and Myotonic Dystrophy type 2 (DM2) (OMIM #602668). DM1 and DM2 are clinically similar, sharing core features such as myotonia, muscle weakness and early onset cataract, but there are some important differences allowing the distinction between the two forms [1, 2].

In this article we aim to provide an updated point-by-point review concisely addressing clinical, molecular, therapeutic, and management key aspects of both DM1 and DM2 in a practical and ready-to-use way. This comprehensive overview will delve into the genetic discoveries associated with both conditions, elaborate on their distinct clinical features, unravel the molecular mechanisms underlying their pathogenesis, and discuss the latest available therapeutic interventions and management strategies.

Additionally, the review will incorporate the most recent guidelines for the management of the myotonic dystrophies, ensuring that the information is up to date, reflecting developments in the field until 2024. This comprehensive exploration seeks to enhance our comprehension of these disorders, facilitating a more informed approach to their clinical management and providing a pocket guide for the clinician dealing with DM patients.

## Clinical features

Even though DM1 and DM2 share common “core” muscular symptoms, such as myotonia, muscle weakness and atrophy and cataract, there are some important differences allowing the distinction between the two forms [2–5]. DM1 is one of the most common rare diseases with an estimated prevalence of 9.27 cases per 100,000, based on a recent meta-analysis performed by Liao and colleagues and including original research articles published, in English, in The PubMed, MEDLINE, Web of Science and Cochrane Library databases till 2022 [6]. DM2, also known as proximal myotonic myopathy (PROMM), shares similarities with DM1 but usually presents in adulthood and with a milder phenotype. In addition, a congenital form of DM2 has never been reported so far [1, 5, 7].

The following table (Table 1) provides a concise overview of the clinical features of DM1 and DM2, highlighting similarities and differences between the two types of myotonic dystrophy. Genetic differences may contribute to the distinct clinical profiles among DM1 and DM2 (see *Molecular Genetics*, Chap. 3). Similarly, DM1 and DM2 clinical similarities may derive from certain common molecular mechanisms underlying the conditions (see *Disease Mechanisms*, Chap. 4).

The disease exhibits a high clinical variability, even among affected family members, and the involvement of different organs and systems, such as heart, eye, endocrine system, genitourinary system, and the central nervous system (CNS). The multisystemic involvement should be regularly screened, although specific guidelines are often lacking. Regarding CNS involvement, a recent study, performed on 163 DM1/2 molecularly confirmed probands, investigated transcranial sonography (TCS) findings in such patients, revealing significant brainstem abnormalities. In DM1, brainstem raphe (BR) hypoechogenicity appeared to be linked to older age, weakness, depression, and fatigue, while an enlarged third ventricle (DTV) correlated with age and disease duration. In DM2, substantia nigra (SN) hyperechogenicity was related to fatigue, and excessive daytime sleepiness with hypoechogenic BR and enlarged DVT. Authors concluded that TCS could be a valuable neuroimaging technique, offering insights into brainstem structures and potentially aiding in understanding the pathogenesis of brain involvement in DM [11]. Additionally, as stressed by Simoncini and colleagues, several approaches have been recently developed to assess CNS involvement in DM1, including traditional psychometric and cognitive tools as well as more advanced psychophysical, neurophysiological, and computerized neuroimaging techniques. These methods aim to enhance the understanding of this aspect of the disease and emphasize its potential as a useful marker for evaluating the effectiveness of future therapeutic treatments [12].

**Table 1:** Clinical and molecular features of DM1 and DM2

CLINICAL AND MOLECULAR FEATURES	DM1	DM2
**Prevalence**	Last esteem: 9.27 cases per 100,000 [6]	Last esteem: 2.29 cases per 100,000 [6]
**Genetics**	● CTG repeat expansion in *DMPK* gene, chr 19q13.3 ● Autosomal Dominant ● Abnormal alleles size: 50 − 4.000 ● Anticipation: + ● Somatic mosaicism: +	● CCTG repeat expansion in *CNBP* gene, chr 3q21.3 ● Autosomal Dominant ● Abnormal alleles size: 75 − 11.000 (average: 5.000) ● Anticipation: not proven ● Somatic mosaicism: +
**Onset**	**Variable** (congenital/childhood/juvenile/adult/late adult)	Typically, **adult-onset**
**Clinical Forms**	**Five clinical forms** [8]: ● Congenital (onset < 1st month, the most severe: polyhydramnios and reduced fetal movements) ● Childhood (1–10 years) ● Juvenile (10–20 years) ● Adult (20–40 years) ● Late Adult (> 40 years: the mildest)	**No clinical classification; absence of congenital form**
**Muscular Symptoms**	● Myotonia (hand, tongue, jaw) [4, 9] ● Weakness and atrophy (long finger flexor; neck flexor; facial muscles; feet dorsiflexors; wrist extensors/flexors) [4, 9] ● Pain (**rarely** reported) [4, 9]	● Myotonia (hand; proximal legs) [10] ● Weakness and atrophy (long finger flexor; neck flexor; thumb flexors; hip flexors/extensors) [10] ● Pain (**frequently** reported) and muscle stiffness [10]
**EMG findings**	Myotonia (wax and waning; **distal**)	Myotonia (waning; **proximal; sometimes not reported**)
**Muscle Histology**	Atrophy and nuclear centralization (mainly **type 1** fibers)	Atrophy and nuclear centralization (mainly **type 2** fibers)
**Systemic Involvement**	Common and more severe than in DM2 [3, 7, 11]	Milder and less common compared to DM1
***Eye Involvement***	● cataract (“Christmas tree” appearance by slit lamp examination), onset around 30–40 years old ● ophthalmoplegia	cataract (posterior subcapsular); sometimes is the first symptom (< 50 years old)
***Cardiac Involvement***	Common, may be significant: ● **mainly** conductive defects ++ ● supraventricular arrhythmias + ● left ventricular dysfunction +	Present but often milder [5]: ● conductive defects + ● supraventricular arrhythmias + ● **mainly** left ventricular dysfunction ++
***Endocrine Involvement***	Hyperinsulinism, thyroid dysfunction, diabetes mellitus, calcium dysregulation, testicular atrophy, possible abnormalities in growth hormone secretion, alopecia	
***Brain Involvement (signs)***	Mild cortical atrophy and white matter abnormalities	
***Brain Involvement (symptoms)***	**100% of patients** [12, 13] ● Cognitive Impairment (from mild to severe) ● Daytime Sleepiness and fatigue	**2/3 of patients** [14, 15] ● Cognitive Impairment (mainly visuospatial and executive dysfunctions) ● Daytime Sleepiness and fatigue
***Reproductive dysfunctions and pregnancy complications***	Higher rates of [16]: ● Fertility issues ● Pregnancy complications (placenta praevia, postpartum haemorrhage; polyhydramnios; miscarriages; perinatal death) ● Delivery complications (preterm or instrumental delivery; non-vertex present)	Higher rates of [16]: ● Fertility issues ● Urinary Tract Infections ● Delivery complications (preterm labour; stillbirth)
***Other***	Increased risk of both benign and malignant tumors [17, 18]	Higher rates of autoimmune diseases (compared both to general population and to DM1) [19, 20]

## Molecular genetics

Myotonic dystrophy type 1 and type 2 are genetic disorders caused by mutations, involving two different genes, both leading to a repeat expansion in a DNA region transcribed into RNA but not translated into protein [21–24].

DM1 and DM2 were considered a single disease until the beginning of 1900, when Steinert and colleagues described the “classic” form of myotonic dystrophy and called it “Steinert disease” or myotonic dystrophy type 1 [25]. Several years later, in 1992, studies revealed that the gene defect responsible for DM1 was the expansion of a CTG repeat in the 3’ untranslated region of *DMPK* (*Dystrophia Myotonica-Protein Kinase*) gene, located on the long arm of chromosome 19 (chr19q13.3) (OMIM #605377) [26–28].

In 1994, Thornton and colleagues described clinical cases in the USA characterized by a “Steinert-like” syndrome with systemic involvement, similar to DM1 for certain clinical features, in which the CTG expansion on chromosome 19q was not detected [29]. Families with a similar phenotype were also described in Italy, in which both DM1 and non-dystrophic myotonia were excluded [30]. Additional studies revealed the unstable tetranucleotide CCTG repeat expansion in intron 1 of *CNBP* (*Cellular Nucleic Acid-Binding Protein*) gene, located on the long arm of chromosome 3 (chr 3q21.3) (OMIM #116955), as the causative mutation for these “Steinert-Like” myotonic dystrophy [31, 32]. In 1999, the International Myotonic Dystrophy Consortium [33] named Myotonic Dystrophy type 2 all the clinical cases linked to *CNBP* tetranucleotide expansion.

Both DM1 and DM2 are inherited in an autosomal dominant manner: each child of an individual with a CTG or CCTG repeat expansion has a 50% chance of inheriting the expansion. For DM2, de novo pathogenic variants have not been reported to date: all affected individuals have had one parent with a CCTG repeat expansion.

### Myotonic dystrophy type 1 (DM1) - *DMPK* gene

The *DMPK* gene contains 15 exons distributed over about 13 kb of genomic DNA on chromosome 19q [34].

The diagnosis of DM1 is established in a proband with identification of a heterozygous pathogenic trinucleotide CTG expansion in *DMPK* gene by molecular genetic testing. The mode of inheritance is thereby autosomal dominant.

Reference ranges for allele sizes were established by the Second International Myotonic Dystrophy Consortium (IDMC) in 1999 (Table 2).

**Table 2:** Reference ranges for allele sizes in DM1

ALLELES SIZE	NUMBER OF DMPK CTG REPEATS	STABILITY/INSTABILITY	DM1 PHENOTYPE
**Normal size**	5–35	Stable	No DM1
**Premutation**	35–50	May be unstable	No DM1
**Expanded 1 (full mutation)**	50–150	Unstable	Mild DM1
**Expanded 2 (full mutation)**	150–1000	Unstable	Classic DM1
**Expanded 3 (full mutation)**	> 1000	Unstable	Congenital DM1

Under 35 CTG repeats, alleles are defined as normal alleles. Individuals with an expansion between 35 and 50 CTG repeats fall in the premutation range and are clinically asymptomatic, but their alleles are at increased risk of expansions in their sibs (“mutable normal, premutation”). Once expanded over the 50 CTG repeats, the disease becomes manifest (“full mutation”; ≥ 50 CTG repeats) [2, 3].

DM1 presents variable expressivity even among the members of the same family. Several studies have demonstrated the correlation between CTG repeat length and disease severity: in general, longer CTG repeat expansions in the *DMPK* gene typically lead to an earlier onset and more severe symptoms in DM1, meanwhile smaller abnormal repeats (less than 100) often correlate with milder or asymptomatic forms [35–38].

In addition, the CTG trinucleotide repeat is unstable during cell division, leading to varying repeat lengths among different tissues (mitotic instability) [38]. This phenomenon, known as somatic mosaicism, is more evident for full mutation alleles and makes it difficult to directly correlate the size of the CTG expansion in one tissue with disease severity [2]. Nevertheless, a subject usually is born with a certain CTG repeat size, which tends to increase over time. The number of trinucleotide repetition present at birth is known as ePAL (Estimated Progenitor Allele CTG Repeat Length) and several studies attempted to estimate it [39].

It must be said that due to somatic mosaicism, even if repeat expansion length is predictive of clinical severity and age of onset, CTG repeat size correlates more significantly with age of onset and disease severity below 400 CTG repeats [40]. In addition, somatic mosaicism makes it impossible to establish a clear correlation between clinical severity and CTG repeat size in tissues other than blood: for example, in muscle cells, despite larger CTG repeats, there is no direct correlation with the degree of weakness. To date, there is no clinical advantage of measuring repeat size in muscle and the genetic test is performed on blood samples [2, 9].

Additionally, a small percentage (3–11%) of DM1 patients contains, in between the pathological expansion, specific variant repeats (such as (CCG)n and (GGC)n repeats) which might influence disease onset and severity [41]. These variant repeat interruptions seem to be associated with later onset, and DM1 families with these variant repeats tend to have less severe symptoms or a milder phenotype [42] (*see Genetic Testing*, Chap. 6).

In myotonic dystrophy type 1, the length of CTG repeats in the *DMPK* gene exceeding 35 repeats tends to be unstable and can expand in length during gametogenesis. This results in the transmission to the offspring of an affected proband of longer trinucleotide repeat alleles that may be associated with a worse disease severity or earlier onset of symptoms than that observed in the parent. This phenomenon is termed “genetic anticipation”.

It is particularly noticeable in cases where the expanded allele is inherited from the mother, leading to congenital DM1 in children. Interestingly, while congenital DM1 is predominantly inherited from the mother, there are instances where anticipation occurs even with paternal transmission [43]. A recent multicenter study on a pediatric cohort reported a paternal transmission rate of 13% [44].

A study involving children of parents with smaller expansions (ranging from 50 to 100 CTG repeats) revealed that those inheriting expanded alleles from their fathers showed a larger increase in CTG repeats compared to those inheriting them from their mothers [45].

The transmission of DM1 is not always characterized by an increase in the CTG repeat size: there are rare occurrences of intergenerational contraction, more commonly observed in paternal transmission [46].

Considering all these aspects, it’s clear how the genetic and clinical variability of DM1 depends on the sex and age of the transmitting parent, on the repetition length, on the presence of repeat interruptions and/or on the degree of somatic instability [37].

Penetrance of DM1 is nearly 100% by age 50 years [3].

### Myotonic dystrophy type 2 (DM2) - *CNBP* gene

In myotonic dystrophy type 2 the diagnosis is established by identification of a heterozygous pathogenic expansion of a CCTG repeat within a complex repeat motif, (TG)n(TCTG)n(CCTG)n, present in the *CNBP* (CCHC-type zinc finger, nucleic acid binding protein; previously known as zinc finger 9 gene, *ZNF9*) gene, located on the long arm of chromosome 3 (chr3q.21). This gene produces a 7-zinc finger domain RNA-binding protein [47].

Alleles presenting less than 30 CCTG are considered stable (normal size), meanwhile in DM2 affected patients the expansion sizes vary from approximately 75 to more than 11,000. Alleles with a number of repeats over 30 but under 75 are considered “mutable normal” (premutation) alleles (∼ 30-∼54 CCTG repeats) or alleles of unknown significance (premutation vs. pathogenic) (∼ 55–74 CCTG repeats). In these last two cases, sequence analysis is necessary to better estimate the size of the CCTG repeat [1, 10, 30]. Healthy individuals typically have interruptions (GCTG, TCTG, or ACTG motifs) within their (CCTG)n repeat, while expanded alleles in DM2 lack these interruptions [48].

As for DM1, DM2 is characterized by somatic mosaicism: the CCTG repeat expansion shows size differences between generations in the same family. In general, the repeat size appears to contract when passed on to the subsequent generation and then to increase in size as the affected individual ages. There is no maternal or paternal preference for contraction or expansion. Even DM2 presents a high inter/intrafamilial variable expressivity. Differently from DM1, anticipation phenomenon is doubtful in DM2, and there is no correlation between onset age and severity, and CCTG repeats length [6]. Nevertheless, the uncertainty surrounding clinical anticipation, congenital forms, and parent-of-origin effects in DM2 prompted further investigations: Gonzalez-Perez and colleagues [18] explored the influence of parent-of-origin on the age of the first DM2-related clinical manifestation. They studied 26 DM2 patients with maternal or paternal inheritance and collected 61 additional patients from the literature. They concluded that maternal inheritance correlated with earlier symptom onset, particularly within the first three decades of life, and increased the risk of cataracts and cardiovascular events as initial DM2 manifestations.

## Disease mechanisms

Individuals affected by diseases due to repeat expansions of DNA show repeat tracts longer and unstable with respect to unaffected people. As previously said, the length dynamic varies through generations, individual life span, among tissues from the same individual, and in a same tissue all along patients’ life, in both mitotic and post-mitotic cells, though disease signs are particularly marked in postmitotic tissues (brain, heart, and skeletal muscle). Pathological expansions generate unusual DNA, DNA-RNA, and RNA structures which interfere with nucleic acid replication, repair, recombination and transcription [49–51].

### Alternative splicing defects

General agreement exists on aberrant alternative splicing (AS) as the main pathomechanism in DM1 and DM2. AS, which modulates the expression of a single gene in different tissues and at different cell stages, is crucial in skeletal muscle for normal myogenesis and adaptation to adult metabolism and functions. Regulatory factors, including RNA-binding proteins (RBPs), are involved in inclusion or exclusion of different exons in each transcript, and Muscle-blindlike (MBNL) family, CUG-BP and ETR-3-like factors (CELF) family and the RNA-binding Fox (RBFOX) are the main splicing regulators in skeletal muscle [52, 53]. MBNL proteins bind to RNA secondary structures [42], particularly MBNL1, which is the most abundant MBNL in adult cardiac and skeletal muscle where it is entrapped in CUG expansion proportionally to the repeat length. Retention of both MBNL and DMPK in the nucleus decreases their functional concentrations in cytoplasm and over time leads to impaired translation and, in addition, to cell toxicity. MBNL2 seems to be more active in the brain tissues. MBNLs also seem to be involved in regulation of mRNA localization and stability [54]. CELF1 (previously called CUGBP1) belongs to the family of CELF (CUGBP, Elav-like family) proteins and binds to single-stranded UG-rich RNA sequences with a lower affinity than MBNL1. Sequestration of MBNL1 induces CELF1 upregulation which, in turn, causes the expression of embryonic variants of transcripts, the main characteristic of spliceopathy in DM1 [55, 56]. MBNL1 and CELF1 act as antagonists on the regulation of pre-mRNA targets as cardiac troponin (*cTNT*), insulin receptor (*INSR*), chloride channel 1 (*CLCN1*) and *MBNL2* [53]. Moreover, in biopsies from DM1 and DM2 patients, accumulation in the nucleus of factors involved in the splicing and maturation of mRNAs (hnRNPs, snRNPs) has been demonstrated [57], which induces cell stress and transcriptome deregulation, This, in turn, induces the dysregulation of stress granule (SG) dynamics regulating the trascriptome during stress. Altered response to stress and abnormal clearance of SGs caused by MBNL1/CELF1 disruption arepossible further elements determining DMs pathology [58, 59].

Even though both expanded CUG (DM1) and CCUG (DM2) RNA interfere with RNA metabolism by titrating MBNL proteins, DM2 exhibits a more favorable course than DM1, hinting at potential modifiers. Sellier et al. [60] revealed that RBFOX1 binds to CCUG, not CUG repeats, competes with MBNL1 for CCUG binding, and partially releases MBNL1 in DM2 muscle cells. Additionally, RBFOX1 expression corrects splicing alterations and rescues defects in a Drosophila DM2 model.

### Repeat-associated Non-ATG (RAN) translation

RAN translation, yielding to the production of non-physiologic peptides /proteins, is toxic to cell. It has been shown that in DMs expanded sequences can be bidirectionally transcribed [61, 62]. In DM1, it is thought that translation of multiple reading frames occurs and produces various proteins, i.e. polyglutamine which accumulates in both the nucleus and cytoplasm leading to apoptosis. Abnormal accumulation of tetrapeptide poly-(LPAC) and poly-(QAGR) were demonstrated in DM2 autoptic brains.

### microRNA dysregulation

microRNAs (miRNAs) modulate gene expression by mRNA degradation, destabilization or translation blocking. They were detected in different models of DM1, some of them possibly being translational repressors of MBNL factors [63, 64], and in peripheral blood of DM1 and DM2 patients, hence might serve as serum biomarkers.

### Circular RNAs (circRNAs)

Circular RNAs (circRNAs), endogenous non-coding and very stable RNAs, are produced by a non-canonical splicing event called backsplicing occurring in a tissue- and cell-specific manner and by the action of cis- and trans-acting factors [65]. circRNAs might contribute to maturation of microRNA (miRNA), to protect target mRNAs from miRNA-dependent degradation, and to complex formation between specific enzymes and substrates. circRNAs are mostly expressed during the differentiation process and predominantly in mammalian brain, striated muscle, and cardiac tissues. Biopsies from DM1 patients showed that upregulated levels of a number of circRNAs were correlated with the severity of the patients’ muscle phenotype [66]. Indeed, circRNA are highly stable and accumulate in non-dividing cells and, due to their resistance to nucleases, they can be detected in serum or plasma and possibly represent useful disease biomarkers.

### Epigenetic modifications

Epigenetic modifications may impact the development or severity of phenotype in DM1. CpGs methylation has been associated with childhood- and juvenile-onset forms of DM1. DNA methylation (DNAme) upstream of the CTG repeats in blood DNA configures as a critical feature of maternally inherited congenital DM1, in addition to repeat length, mother’s age and the clinical features. Légaré and colleagues analyzed the DNAme levels of the *DMPK* gene in a large cohort of DM1 adult patients in order to check for possible links with muscular and respiratory impairments [67] and they did find association with several muscular and respiratory parameters, which could help understanding the phenotypic variability among DM1 patients. DNAme might be related to the cognitive dysfunction often reported in DM1 [9], as assessed in 115 affected adults on a 9-year follow-up period, independently from the CTG repeat length. Further studies are needed to clarify this relationship, anyway DNAme level may become a biomarker for monitoring DM1 disease progression, allowing a more reliable prognosis for the cognitive, muscular, and respiratory dysfunctions. Unfortunately, up to date no study on the association of DNAme of *CNBP* gene and the different DM2 clinical subtypes has been performed.

### Cellular nucleic acid binding protein (CNBP)

Cellular nucleic acid binding protein (CNBP) is capable of binding to human both single stranded DNA and RNA molecules and regulates the expression of multiple genes by activating or repressing their transcription [68]. A mouse model overexpressing *CNBP* increased CNBP and ClC-1 levels and rescued the phenotype, suggesting that, in addition to RNA gain of function, a loss of CNBP may contribute to DM2 pathology [69]. Nevertheless, an in vitro model demonstrated that pre-mRNAs containing large CCUG expansions are normally processed, that the expansions do not decrease CNBP expression at the mRNA or protein level, and that foci are enriched for the CCUG expansion, therefore different mechanisms could act at transcriptional level with respect to DM1 [70].

### CLCN1 and SCN4A mutations as possible modifier

Mutations in *CLCN1* and *SCN4A* genes are associated with non-dystrophic myotonic disorders (skeletal muscle channelopathies); double-troubled cases of patients with DM2 from Germany and Italy suggest a possible modifying role in the development or severity of myotonia and/or muscle pain by enhancing the myotonia and/or muscle pain in some patients with DM2 [71–73].

## Animal and cellular models

Numerous animal models have been developed to study germline and somatic instability and try to replicate the multisystemic pathophysiology shown by patients [74, 75]. Historically, the study of myotonic dystrophies has focused more on DM1 than on DM2 with the production of mouse models that reproduce the phenotypic characteristics of DM1 in different tissues up to the multisystemic expression typical of the disease with results complementary to each other [76–78]. In DM2, haploinsufficiency of *Znf9* gene in mice showed muscle wasting, myotonic discharges, heart abnormalities and great decrease of Clc1 channels suggesting that haploinsufficiency contributes to disease phenotype in mice [69].

Animal models other than mouse models were used to better understand the mechanisms that affect both the germline and the somatic cells and contribute to DM1 pathogenesis. *Caenorhabditis elegans* showed that in DM1 the RNA interference machinery is important in causing RNA toxicity and disease phenotypes [79]. In *Drosophila melanogaster* DM1 models the severity of the phenotype is correlated with the length of the repeat expansion as in DM1 patients. These models have been useful to study CUG repeat toxicity and alternative splicing due to the presence of both muscle and cardiac pathology [80]. *Drosophila* has been also used as a model of DM2: the flies showed RNA foci and missplicing, impairment of muscle, eye and heart, accelerating the knowledge of the pathways deregulated in DM2 [81].

*C. elegans*,* Drosophila* and zebrafish models are compatible with high-throughput screening and are used to test multiple molecules as they are models faster to reproduce than mice. Animal models are also useful for drug repurposing: it has been demonstrated that verapamil improves survival in a bichannelopathy mouse model which represents the combinatorial effect of *Clcn1* and *Cacna1s* missplicing typical of DM1 patients [82], and that the administration of erythromycin improves splicing abnormalities in DM1 mice [83].

The identification of numerous pathways involved in myotonic dystrophies has led to the examination of the individual mechanisms involved in pathogenesis to better identify causes and possible therapies. In this scenario, cellular models have been very useful to dissect molecular mechanisms and evaluate therapeutic formulations. From human primary cells derived from patients’ biopsies to immortalized cell lines and human induced pluripotent stem cells, all of these cellular models had an important role in disease understanding [84]. Recently, stem cells have been used to assess the therapeutic potential of genome therapy in DM1 mediated by both CRISPR-Cas9 and TALEN [85, 86]. Indeed, it has been recognized that senescence influences the pathophysiological mechanisms of DM1 in reducing the regenerative capacity of muscle tissue [87]. A senescent molecular signature has been identified in DM1 myoblasts and was demonstrated that the use of specific drugs that induce apoptosis of senescent cells restore the regenerative capacity of myogenic cells [87]. 3D cultures as cortical organoids have also been used to model DM1 neuropathology suggesting that neurodevelopmental disorder-associated pathways could be involved in early onset DM1 [88].

## Genetic testing

The diagnosis of both types of myotonic dystrophies represents a challenge in terms of determining the size of the disease expansion [89]. Conventional methods are based on gDNA PCR amplification of the region harboring the CTG or CCTG repeats in order to first distinguish healthy from pathological alleles, and hence to evaluate expansions exceeding the normal range. A short-range PCR, followed by separation on agarose gel [90], allows the identification of normal alleles differing at least for 15–20 bp, whereas a more sensitive method based on bidirectional triplet-repeat primed amplification (TP-PCR) of fluorescence-tagged fragments, followed by capillary electrophoresis, enhances resolution of very similar lengths [91]. Data essentially come from analysis on DM1 patients. TP-PCR restriction lies in capillary separation performance, reaching the upper limit of detection of 200(CTG). A further step in sizing the pathologic alleles has been traditionally represented by southern blot gel separation of amplicons generated by XL-PCR, enabling the identification of 8–10 kb expansions, in both types of DMs.

In DM1, a relevant cue to consider is the possible, yet rare (3–11% patients) [41] presence of expansions interrupted by non-canonical triplets (CCG, GGC, CTC, CAG) associated with a higher stability of the repeat number between generations and decreased individual somatic mosaicism, leading to milder phenotypes and later age of onset [92, 93]. These interruptions can stabilize the expansion rate, either by increasing DNA strand stability during cell divisions, or by producing conformational changes of the variant RNA species with effects on their toxic gain-of-function, subsequently delaying symptom onset and progression. However, there’s debate about how significantly variant repeats affect the toxic function of expanded RNAs [94]. The presence of variant repeats can lead to false negatives in standard PCR-based approaches, so that TP-PCR is recommended for DM1 testing [95]. Even though, up to now, only TP-PCR can be useful to detect interruptions within the 5ʹ and 3’ end of the CTG repeats, it cannot identify interruptions sited in the middle and give the exact number and type of interruptions.

In addition, assessment of the level of mosaicism may help in predicting disease progression [91]. As far as mosaicism, since the repeat length is depending from the patient’s age, evaluation of the CTG repeat inherited from the affected parent, the progenitor allele length (ePAL) which is independent from age of sampling [96], may help in predicting the age at onset and in improving the prognostic ability. A method has been proven to fit this aim, small pool PCR (SP-PCR) [97], as it uses 0.5 to 200 genome equivalents thus allowing the identification of products derived from single-input molecules.

All methods described so far show limitations to their effectiveness, especially in cases of large and complicated repeats. Therefore, a faster and more sensitive method was needed. On the basis of direct sequencing, it was developed an amplification-free targeted longread sequencing, and it was assayed on *DMPK* expanded regions. It circumvents PCR amplification and relies on CRISPR-Cas9 technology to simultaneously analyze the size and sequence targeted repeats. In a pilot study on DM1 patient samples, it has been proven to detect CTG repeat expansions and somatic mosaicism [89], together with interruptions, regardless of the size of the CTG repeat expansions. A new pre-packaged and ready to use genetic test has been developed and validated for DM2, based on the combination of Long-PCR and Southern Blot Analysis (SBA) [98]. SBA associated with field-inversion electrophoresis (FIGE) or Tetraplet-Primed PCR (TP-PCR) has also been used to improve the molecular diagnosis [99]. However, because of a high incidence of false positive results [100], this method was suggested only as supportive to overall diagnostic flowchart, which includes skeletal muscle histopathology and in situ hybridization (ISH) on muscle sections. Indeed, whereas in DM1 muscle biopsy is unnecessary for diagnostic purposes, in DM2 it might be useful since the clinical and instrumental signs are milder and less suggestive. Patients are possibly addressed to further DM2 molecular analysis on the basis of characteristic histopathologic features: preferential type 2 fiber atrophy in DM2 and absence of type 1 fiber atrophy; central nucleation selectively affecting type 2 fibers and nuclear clumps expressing fast myosin isoform (type 2 fiber) [101].

## Management

Currently, no curative treatment has been formally approved for patients with DM. Management remains based on supportive care, including vigilantly monitoring for the onset of disease-related complications [3, 7, 11]. The management strategies for DM2 mirror those for DM1, with few treatments tailored specifically for DM2. Strategies to manage multisystemic DM 1 and 2 complications are summarized in Table 3. Prioritizing genetic counseling, strategies to maintain functional independence, addressing the disease’s multisystemic involvement, and providing symptomatic relief stand as key in managing DM patients. While a neuromuscular specialist’s referral and monitoring remain essential, the intricate nature of the condition necessitates a multidisciplinary approach. Collaborating with cardiologists, pulmonologists, ophthalmologists, physiatrists, gastroenterologists, endocrinologists, and geneticists is crucial to ensure comprehensive and optimal clinical care for DM patients. Attending treatable multisystemic features and other symptomatic aspects not only enhances patients’ quality of life but also extends their lifespan [102]. Diminished life expectancy in DM1 is primarily due to respiratory failure, cardiac rhythm disturbances, and an elevated risk of malignancies [3, 103]. Even mildly affected individuals face potential risks of sudden cardiac death or respiratory failure during general anesthesia, underscoring the critical importance of early diagnosis and holistic care for individuals with DM1 [104].

Another important aspect in the management of DM patients is the “End-of-life management”: it should involve palliative care services to maximize quality of life and address caregiver burden, nevertheless to support both patients and their families through the challenges of progressive neuromuscular diseases, offering guidance on care plans and transitions in care as the disease progresses [3, 7]. Willis and colleagues [105] recently outlined guidelines for palliative care in patients with myotonic dystrophy type 1, emphasizing the importance of early palliative care introduction, particularly when significant swallowing and cardiorespiratory complications arise, as these are major contributors to mortality. It suggests a traffic light system for triaging patients based on their condition’s severity, recommending referral to palliative care specialists at appropriate stages. Advance care planning (ACP) is highlighted as crucial, allowing patients to record their preferences for end-of-life care, facilitating better outcomes and access to preferred care settings. Authors also discuss about the necessity for honest discussions about end-of-life care, especially considering potential cognitive issues in DM1 patients, and recommends regular review of ACPs to ensure patient preferences are respected.

**Table 3:** Management strategies in patients with myotonic dystrophy type 1 and 2

ORGAN/SYSTEM/CLINICAL ISSUE	MANAGEMENT STRATEGIES
**Musculoskeletal**	● For severe, disabling myotonia impacting daily activities, mexiletine (150–200 mg three times a day) is recommended as a first-choice anti-myotonic drug, with prior and regular ECG monitoring. ● Regular low or moderate aerobic exercises, inclusion of physical, occupational, and speech therapists ● Access to mobility aids or orthopedic treatment for preserving functional independence. ● Pain in skeletal muscles is a common feature in DM2 and less so in DM1, aggravated by cold temperatures, though its precise mechanism is unknown. Various pain medications like mexiletine, gabapentin, NSAIDs, low-dose thyroid replacement, steroids, and tricyclic antidepressants have been explored, albeit none consistently effective.
**Eye**	● Annual ophthalmologist check-ups with slit lamp examination. ● Surgical treatment for cataracts or severe ptosis if symptoms interfere with daily activities. ● Optical coherent tomography (OCT) for visual impairment to avoid misdiagnosis of treatable conditions.
**Heart**	● Annual ECG monitoring, with cardiologist referral for symptomatic patients or those with abnormal findings. ● A 24-h Holter ECG for symptomatic subjects, and echocardiography at diagnosis and every 3–5 years, or more frequently if initial findings are abnormal. ● Use antiarrhythmic drugs cautiously and consider implantable cardioverter defibrillators for ventricular tachyarrhythmias and anticoagulants for atrial fibrillation/flutter.
**Respiratory System**	● Baseline and annual respiratory examination with pulmonary function tests. ● Monitor for recurrent respiratory infections in patients with dysphagia, impaired cough, and lung clearance, providing cough assistance techniques and devices. ● Recommend vaccination against SARS-CoV-2, influenza, and pneumonia.
**Endocrine System and Metabolism**	● Assess hormonal status, lipid profile, and glucose metabolism at baseline. ● Monitor HbA1c and fasting serum glucose annually. ● Follow American Diabetes Association (ADA) or other relevant guidelines for glucose metabolism disorder management, preferring metformin for impaired glucose metabolism. ● Regularly assess thyroid hormone status and lipid profile every 3 years, prioritizing cholesterol-lowering agents other than statins (e.g. Ezetimibe). ● Refer patients with gonadal dysfunction or fertility issues to subspecialists.
**Central Nervous System**	● Inform patients and caregivers about DM1 as a brain disorder. ● Routinely assess for cognitive, behavioral, or psychiatric impairments, with neuropsychological assessment post-diagnosis. ● Consider referral to mental health specialists and specific treatments. ● Regularly obtain fatigue and sleep scale scores and treat excessive daytime sleepiness (EDS) with modafinil, methylphenidate, or caffeine/theobromine. ● Cognitive behavioral therapy (CBT) for severely fatigued patients.
**Gastrointestinal System**	● Screen for dysphagia, dysphonia, and weight loss, introducing dietary modifications and “safe swallowing techniques” if needed. ● Consider gastrostomy for severe cases. ● First-choice therapy for GI symptoms includes dietary modifications; pharmacotherapy for persistent symptoms. ● Monitor for life-threatening complications like aspiration and pseudo-obstruction. ● Refer to specialists for acute abdomen symptoms to exclude serious conditions.
**Anesthesia**	● High risk of sensitivity to anesthetic drugs, with detailed preoperative evaluation and peri/postoperative cardiopulmonary monitoring recommended. ● Prefer local anesthesia; avoid anticholinesterase drugs, depolarizing muscle relaxants, and inhalation anesthetics for general anesthesia due to exacerbation risks of muscle weakness and myotonia.
**“End of Life”**	Importance of early palliative care introduction, particularly when significant swallowing and cardiorespiratory complications arise, in order to (1) maximize quality of life and address caregiver burden; (2) support both patients and their families through the challenges of progressive neuromuscular diseases.
**Cancer risk**	Increased malignancy risk in DM1 patients requires the strict adherence to the general cancer screening guidelines, especially for ovarian, endometrial, colon, brain, skin and thyroid neoplasms that are more frequent in DM1 compared to the general population.
**Pregnancy and delivery complications**	Increased pregnancy and delivery related risks require a multidisciplinary approach to DM women (obstetrician, anesthesiologist, neonatologist, geneticist, midwife, physiotherapist)

## Therapies under investigation for DM1

Myotonic Dystrophy Type 1 presents a significant challenge in genetic disease treatment, characterized by its complex pathogenesis and wide range of clinical symptoms. The pursuit of effective therapies for DM1 has been a dynamic and evolving journey, with recent years marking a period of unprecedented progress. This chapter delves into the advancements across three primary therapeutic categories (Table 4): small molecules, nucleic acid-based therapies, and genome/transcriptome engineering, which collectively offer a multifaceted approach to combating DM1.

### Small molecules: bridging the gap to market authorization

The exploration of small molecules in DM1 treatment leverages existing pharmacokinetics (PK) and pharmacodynamics (PD) profiles, facilitating a swift transition from theoretical models to clinical applications (Table 3). This strategy not only reduces costs and risks but also accelerates the journey towards market authorization. Despite varying efficacy among candidates, the repurposing of drugs for DM1 signifies a critical step forward, demonstrating the potential of small molecules to modulate disease pathways and alleviate symptoms.

### Nucleic acid-based therapies: harnessing precision medicine

Nucleic acid-based therapies (NATs) have emerged as a cornerstone of precision medicine in DM1, driven by their capacity to target the genetic underpinnings of the disease. Through mechanisms like RNA interference (RNAi) and antisense oligonucleotides (ASOs), NATs offer a targeted approach to modulate gene expression, mitigate toxic RNA accumulation, and correct aberrant splicing patterns. The specificity of NATs enables the precise modulation of gene or gene product expression, positioning these therapies at the forefront of genetic disease management.

The development of RNAi techniques exploits the vast transcriptome for gene expression regulation [106]. By integrating into the RNA-induced silencing complex (RISC) and targeting complementary mRNA sequences, RNAi facilitates the degradation or translational blocking of target mRNAs [106]. This endogenous pathway, augmented by exogenous compounds, offers a promising avenue for DM1 treatment, addressing the disease’s molecular abnormalities directly. The MARINA trial (NTC05027269; results unposted), sponsored by Avidity Biosciences, is currently testing a conjugate of siRNA targeting *DMPK* RNA with a monoclonal antibody against transferrin receptor 1 (TfR1) [107]. Individuals with DM1 are undergoing intravenous treatments to introduce siRNA directly into their muscle cells. This early-phase clinical trial, while not aimed at evaluating functional outcomes, still assesses critical clinical indicators, including mobility, muscle strength, and patient self-reported outcomes. An extended study (MARINA-OLE) has been introduced for participants who have already completed the initial trial, where they will continue to receive AOC 1001 for an added two years (NCT05479981; results unposted). Achievements for AOC 1001 include receiving orphan drug status and fast-track designation from regulatory bodies. The latest report announced mid-study positive results in achieving specific RNA targeting in muscles, significantly reducing DMPK levels and enhancing alternative splicing in muscle-related genes across all participants, with notable clinical improvements observed in certain individuals at the higher dose level.

Conversely, the ongoing ACHIEVE trial is testing an ASO, named DYNE-101 (NCT05481879). This trial recruiting a big number of patients will start soon also in Europe. ASOs are brief synthetic RNA strands, ranging from 8 to 50 bases, that initiate the degradation of target RNA via RNase H [108]. ASOs have gained attention in neurology, leading to FDA approvals for therapies like Spinraza for spinal muscular atrophy [109], tofersen for Amyotrophic Lateral Sclerosis [110], eteplirsen for Duchenne muscular dystrophy [111] and inotersen for TTR amyloidosis [109]. However, their effectiveness is limited by challenges in distribution and bioavailability, highlighting the importance of precise delivery methods. The approach for DYNE-101, also known as the FORCE-DMPK candidate, employs the proprietary FORCE™ platform by Dyne Therapeutics, utilizing TfR1’s expression on muscle cells for targeted delivery [112]. This strategy involves attaching an ASO to monoclonal antibody fragments that bind the transferrin receptor, aiming to decrease *DMPK* RNA within the nucleus through RNase H cleavage. Demonstrated efficacy in reducing harmful *DMPK* RNA and improving genetic markers in a novel mouse model has shown promise, alongside a favorable safety profile in nonhuman primate studies [113]. The ACHIEVE trial is now assessing the compound’s safety and effectiveness in human subjects. Ionis previously tested baliforsen (ISIS 598769), an ASO targeting *DMPK* mRNA, in DM1 patients. The drug was generally well tolerated but skeletal muscle drug concentrations were below levels predicted to achieve substantial target reduction, so the study was discontinued [114].

Another interesting class of ASOs is represented by antimiRs, a class of chemically engineered ASOs complementary to their cognate targets miR-23b or miR-218 [115]. The approach is based on the enhancement of the endogenous expression of MBNL1 and MBNL2, to compensate for functional depletion by sequestration. ARTHEx Biotech developed ATX-01, an antimiR oligonucleotide targeting miR-23b, which will shortly be evaluated in the Phase I-IIa ArthemiR™ trial for the treatment of DM1. ATX-01 has received Orphan Drug Designation for ATX-01 in DM1 from the US and European authorities. Recently, a preclinical study by Gonzalez-Martinez and colleagues [116] investigated the therapeutic potential of peptide-conjugated antimiRs in treating DM1 by enhancing the expression of MBNL1, a protein sequestered by toxic RNA in DM1. AntimiRs targeting miR-23b and miR-218 were conjugated with cell-penetrating peptides (CPPs) to improve delivery to affected tissues. Experiments demonstrated significant increases in MBNL1 levels in DM1 cells and mouse models, indicating a promising approach for overcoming delivery challenges in DM1 therapy. Toxicologic assessments showed no signs of toxicity, highlighting the potential of CPP-PMO antimiRs as effective treatments for DM1.

### Genome/transcriptome engineering: pioneering genetic correction

The advent of CRISPR/Cas9 technology has revolutionized the field of genome engineering, providing an unparalleled tool for precise gene expression regulation and editing [117]. In DM1, CRISPR/Cas9 enables the direct targeting and removal of pathogenic expansions, offering a potential cure by addressing the disease’s root cause [118]. The application of vectorized AAV administration for CRISPR/Cas9 delivery further exemplifies the innovative approaches being explored to harness gene editing in therapeutic settings.

The efficacy of molecular therapies is intricately linked to the challenges of delivery, necessitating innovative strategies to ensure optimal cellular uptake. Viral vectors, particularly adeno-associated viruses (AAVs), have become the delivery method of choice, capitalizing on their ability to infect both dividing and non-dividing cells and ensure stable gene expression [119]. The exploration of AAV serotypes and capsid engineering aims to enhance tissue specificity, minimize immunogenicity, and maximize therapeutic potential. AAV serotype 9 (AAV9), isolated from human liver tissue, was proved to be able to reach the MNs of the spinal cord, the cortical neurons and the deep structures of the brain, due to its unique ability to cross the blood-brain barrier [120–123]. One example of AAV9-based gene therapy is onasemnogene abeparvovec (trade name Zolgensma), approved by the FDA in 2019 for the treatment of SMA [124].

Génethon is studying an approach involving CRISPR-Cas9 from Staphylococcus aureus for targeting *DMPK* CTG expansions (Table 4) [125]. LocanaBio is conversely working with AAV9 vectors carrying a modified Cas9 aimed at CUG repeats that are showing promising results in the reduction of harmful RNA build-up, improvement in splicing, and alleviation of DM1 symptoms in muscle cells and mice, moving towards trials in nonhuman primates [126, 127].

**Table 4:** Emerging therapeutic approaches for DM1

AGENT CLASS	DRUG	MECHANISM OF ACTION	CLINICAL TRIAL STAGE	DEVELOPER	RESULTS FROM CLINICAL TRIALS	Ref.
**Small Molecules**	***Tideglusib***	GSK3b inhibitor reducing toxic DMPK RNA and normalizing CUGBP1 downstream targets	Phase II/III	AMO Pharma	Improved muscle weakness, myotonia, CNS and clinical neuromuscular symptoms in most patients; favorable PK profile	[128]
	***Mexiletine***	Blocks sodium channels to reduce/prevent myotonia	Phase III	Lupin	Positive effect on handgrip myotonia in ambulatory patients; no significant benefit on 6MWT; downregulates DMPK mRNA levels	[129]
	***Pitolisant***	Antagonizes histamine H3 receptors to treat excessive daytime sleepiness	Phase II	Harmony Biosciences	Evaluation for excessive daytime sleepiness in DM1 patients	NCT04886518: results unpublished
	***Metformin***	Biguanide antidiabetic drug rescuing multiple DM1 phenotypes	Phase III	/	Statistically significant improvements in mobility and total mechanical power during gait in more than 50% of patients	[130]
	***MYODM™***	Combination of theobromine and caffeine	Commercialized	Myogem Health Company	Significant decrease in Epworth’s sleepiness scale and increase in 6MWT results; improvements in quality of life in adult males with DM1	[131]
	***Erythromycin***	Decreases foci formation and rescues missplicing in DM1 cell and mouse models	Phase II	/	Recruitment complete; specifics on results not provided	[132]
**Nucleic Acid-Based Therapies**	***IONIS 486,178 & IONIS 877,864***	Target 3’-UTR of DMPK for RNase H-mediated degradation; C16-HA conjugation for improved internalization	Preclinical	IONIS Pharmaceuticals	Reduced DMPK expression, improved therapeutic activity in skeletal muscle and heart, corrected aberrant splicing in neural cells; C16-HA not effective in brain	[52, 53, 133–135]
	***ATX-01***	Enhances expression of MBNL proteins by blocking natural miRNAs	Preclinical	ARTHEx Biotech	Increased MBNL protein levels, improved molecular and functional phenotypes in disease models	
	***AOC 1001***	siRNA conjugate targeting DMPK RNA with monoclonal antibody against TfR1	Phase I/II (MARINA trial)	Avidity Biosciences	Successful targeted delivery and meaningful DMPK reduction; early signs of clinical activity (myotonia improvement)	NTC05027269; results unpublished
	***DYNE-101***	ASO reducing DMPK RNA levels in nucleus by RNase H cleavage, conjugated with TfR1-binding fragments for muscle delivery	Phase I/II (ACHIEVE trial)	Dyne Therapeutics	Robust reduction of toxic DMPK RNA, reversal of myotonia, favorable safety profile	NCT05481879; results unpublished.
	***NTC0231***	PNA sequence targeting pathogenic CUG expansions	Preclinical	NeuBase Therapeutics	Long-lasting correction of misspliced transcripts and broad tissue distribution in mouse models	[136]
	***EDODM1***	Peptide-conjugate oligonucleotide blocking interaction of MBNL1 with toxic RNA	Preclinical	PepGen	Sustained correction of missplicing events and complete rescue of myotonia phenotype in mouse models	[137]
	***ENTR-701***	PMO ASO type, conjugated to cyclic cell-penetrating peptides, blocking CUG repeats in an allele-specific manner	Pre-IND	Entrada Therapeutics	Reduction of nuclear foci and correction of aberrant splicing events in cell and murine DM1 models	[138]
	***CPP-PMO AntimiRs targeting miR-23b and miR-218***	Enhance MBNL1 expression by inhibiting miRNAs that sequester MBNL1	Preclinical	/	Demonstrated increased MBNL1 levels in DM1 cells and mouse models; no toxicity observed in toxicological assessments	[106]
**Genome/Transcriptome Engineering**	***CRISPR/*** ***Cas9***	Adapts CRISPR/Cas9 for removing expansions at the DNA level, preventing transcription, or targeting degradation of toxic RNA	Preclinical	Various (Génethon, LocanaBio)	No clinical trial yet; progressing to preclinical stages with vectorized AAV administration. Génethon strategy has no new data; LocanaBio shows promise with dose-dependent reduction of toxic RNA foci, rescued missplicing, and recovery of muscle weakness	[125, 127]
	***LocanaBio (PIN-dCas9)***	AAV9-vectors encoding PIN-dCas9 and a single-guide RNA targeting CUG repeats to degrade toxic CUG RNA molecules preferentially	Lead Selection & Optimization	LocanaBio	Dose-dependent reduction of toxic RNA foci, redistribution of MBNL1, rescued missplicing, reduced myotonia, and muscle weakness recovery in treated DM1 patient muscle cells and HSALR mic	[126, 127]
**Senolytics**	***Elimination of Senescent Muscle Stem Cells***	Targeting and eliminating senescent muscle stem cells to restore myogenesis	Preclinical	/	Suggested restoration of myogenesis and improvement in muscle function	[87]

## Conclusion

This comprehensive update review has elucidated various aspects of Myotonic Dystrophy type 1 and type 2, ranging from genetic mechanisms and molecular diagnostics to emerging therapeutic strategies. The exploration of disease modifiers and animal models has deepened our understanding of pathogenesis. We discussed advances in antisense oligonucleotide therapy, gene replacement approaches, and the potential of CRISPR/Cas9 technology. As the DM1 treatment landscape continues to evolve, the integration of small molecules, NATs, and genome engineering represents a comprehensive approach to tackling the disease. However, the journey from discovery to clinical application is fraught with challenges, including the need for targeted delivery, the mitigation of off-target effects, and the management of immune responses. The ongoing research and development efforts underscore a commitment to overcoming these obstacles, paving the way for a new era of DM1 therapy. Continued research into novel therapeutic modalities and personalized medicine holds promise for improving outcomes in patients with Myotonic Dystrophy type 1 and 2.
